# Physicochemical and microbiological characteristics of kitoza, a traditional salted/dried/smoked meat product of Madagascar

**DOI:** 10.1002/fsn3.1122

**Published:** 2019-07-15

**Authors:** Angela Ratsimba, Danielle Rakoto, Victor Jeannoda, Herizo Andriamampianina, Régine Talon, Sabine Leroy, Joël Grabulos, Elodie Arnaud

**Affiliations:** ^1^ Faculté des Sciences Université d'Antananarivo Antananarivo Madagascar; ^2^ Université Clermont‐Auvergne‐INRA, MEDIS Clermont Ferrand France; ^3^ CIRAD, UMR QualiSud Montpellier France; ^4^ Qualisud, Univ Montpellier, CIRAD, Montpellier SupAgro Univ d'Avignon, Univ de La Réunion Montpellier France

**Keywords:** beef, drying, kitoza, polycyclic aromatic hydrocarbons, pork, smoking

## Abstract

Kitoza samples collected from producers in Madagascar were analyzed for their physicochemical and microbial properties. Lactic acid bacteria and coagulase‐negative staphylococci were the two codominant populations with average counts of 6–7 log cfu/g. Good hygienic practices were sometimes lacking but samples were not contaminated with *Salmonella*, *Clostridium perfringens*, and *Bacillus cereus* and only once with *Listeria monocytogenes*. *Staphylococcus aureus* was found occasionally with higher counts in salted/dried products than in salted/smoked products. Moisture, protein, fat, and salt contents varied considerably and were on average 41.5, 43.5, 14.3, and 3.3 g/100 g, respectively, and water activity was 0.893 on average. Smoked kitoza showed higher moisture content compared to dried kitoza. Most of the smoked kitoza had a water activity higher than 0.9 which is not in accordance with their storage at ambient temperatures. Benzo(a)pyrene content was above 2 µg/kg in 11 out of 30 smoked samples (17 ± 16.5 µg/kg on average).

## 1 INTRODUCTION

Kitoza is a traditional Malagasy meat product that plays an important role in the daily diet of Malagasy people. The strips of meat (beef mainly but also pork) are mixed with salt and spices, mostly garlic, pepper, and ginger. Then, they are sun‐dried and/or smoked above the fire in the kitchen until consumption or in smoking units. Kitoza is stored at ambient temperatures. It is eaten raw or fried as a snack or included in cooked dishes. Kitoza is no longer only prepared at household level, and it is also produced by butchers and a few small factories. The acceptance study reported in a previous paper (Pintado et al., 2016) showed that kitoza could also be found acceptable by consumers in Portugal and other European countries. In Madagascar, where chilling processes are still expensive and not guaranteed, meat products that remain stable at ambient temperature processed with methods easy to implement, such as kitoza, have great potential.

Kitoza belongs to the wide range of salted/dried/smoked meat products (Santchurn, Arnaud, Zakhia‐Rozis, & Collignan, 2012). Among those made from noncomminuted beef, biltong is the most similar to salted/dried beef kitoza. Biltong is commonly known as a ready to eat salted/dried meat product made from beef but also from game and ostrich meat and is very popular in South Africa (Jones, Arnaud, Gouws, & Hoffman, 2017). It however differed from it as no vinegar is used in the formulation which may have an impact on its shelf life. Kundi is produced by smoking fresh beef, camel, or horse meat in the northern part of Nigeria but the meat can also be salted before smoking (Alonge, 1987) and thus resembles salted/smoked beef kitoza. Unam Inung in Nigeria and Boucané in French overseas department Reunion Island are other salted/smoked meat products made from strips of pork belly (Poligne, Collignan, & Trystram, 2001). As for kitoza, they are traditionally smoked by direct hot smoking of meat strips hung over a fire. Smoking contributes to the shelf stability of the product as it contributes to the reduction of moisture and water activity (*a*_w_) and due to the antioxidant and bactericidal effect of smoke compounds (Sikorski & Sinkiewicz, 2015). However, it may lead to high contamination by polycyclic aromatic hydrocarbons (PAHs). For benzo(a)pyrene (B(a)P), one of the PAHs under regulation in European regulation 835/2011 (European Commission, 2011) which limit is set to 2 µg/kg, values of up to about 35 µg/kg have been reported in smoked meat products around the world (Ledesma, Rendueles, & Diaz, 2016). There are few studies in which meats or fishes processed under direct smoking were assessed for PAH content. Poligne et al. (2001) reported B(a)P content of 6.9 µg/kg in boucané. Other data reported between 20 and 67 µg/kg in kundi (Alonge, 1987), 7.9 and 3–38 µg/kg, respectively, in smoked/dried bush meat (antelope) and fish (Akpambang et al., 2009), and 36.9 μg/kg in ham (Wretling, Eriksson, Eskhult, & Larsson, 2010).

The aim of this study was to assess the microbial quality of kitoza currently produced in Madagascar according to the raw material used (beef or pork) and processing (salted/sun‐dried or salted/smoked) as well as its physicochemical characteristics that are related to the shelf life and its chemical safety.

## 2 MATERIALS AND METHODS

### 2.1 Collection and preparation of samples

A total of 60 kitoza samples (30 beef and 30 pork) were collected from butchers, small factories, and from households in Antanarivo, Madagascar, over a period of 2 months (June and July 2011). For each type of meat, half of the kitoza samples were salted/dried and half were salted/smoked according to the producers. The samples, which each comprised 2–3 strips, weighed at least 600 g each. The strips comprising each sample were cut into cubes of about 1 cm^3^ and mixed. Half the cubes were ground for physicochemical analysis, and the remaining cubes were used for microbiological analyses.

### 2.2 Proximate

Moisture content was determined by placing 10 g of sample at 103°C and atmospheric pressure until stable weight was reached (24 hr). Protein content was determined from the nitrogen content using the Kjeldahl method and a conversion factor of 6.25. Lipid content was determined using the method of Folch, Lees, and Stanley (1957). Briefly, a 20 g sample was stirred with 100 ml of chloroform/methanol (2/1, v/v) for 1 hr. The mixture was filtered over glass fiber, and the filtrate was placed in weighed decantation flasks with 60 ml of salted acidified water (9 g/L NaCl and 0.01 N HCl). After 8 hr of decantation, the bottom phase was removed and placed in round bottom flasks and 40 ml of chloroform was added to the upper phase remaining in the decantation flask. After one more night of decantation, the bottom phase was added to the previous one and evaporated under vacuum (Laborota 4000). The round bottom flask was then placed in a desiccator overnight before weighing.

### 2.3 Salt content

Salt content was determined with a Model 926 Chloride Analyzer (Sherwood Scientific) after a 2‐hr cold extraction in 0.3 N nitric acid.

### 2.4 Water activity

Water activity was measured at 25°C using a fast‐lab water activity meter (GBX).

### 2.5 pH and titratable acidity

pH and titratable acidity were measured with a TitroLine^®^ easy titrator (SI Analytics GmbH) after homogenization of a 3 g sample with 27 ml of distilled water for 30 min.

### 2.6 D‐ and L‐lactic acid contents

To determine D‐ and L‐lactic acid contents, enzymatic kits (Enzytec™, SCIL Diagnostics GmbH) were used on filtered extracts (porosity ø 25 µm). These extracts were obtained by treating the samples with Carrez reagents to precipitate proteins. A 2.5 g of sample was placed in a 50‐ml vial containing 30 ml of distilled water, and then 2.5 ml each of Carrez solutions I (3.6% w/v C_6_FeK_4_N_6_, 3H_2_O) and II (7.2% w/v ZnSO_4_, 7H_2_O), and 5 ml of 0.1 N sodium hydroxide were added successively before completing to 50 ml with distilled water.

### 2.7 Phenol content

Total phenol content was determined according to AFNOR (1996). Briefly, phenolic compounds of the samples were extracted in ethanol. A colored complex of phenols and 4‐amino‐antipyrine was formed in the presence of potassium ferricyanide and separated using chloroform. The absorbance was read at 455 nm. Total phenol content was calculated using a calibration curve of C_6_H_5_OH.

### 2.8 Polycyclic aromatic hydrocarbons

For PAH extraction, 1 g of sample was weighed in a vial and saponified with 3 ml of potassium hydroxide 1 M in ethanol at 80°C under stirring for 30 min. The slurry was cooled and extracted with 3 ml of cyclohexane at 80°C and under stirring for 5 min. After cooling, 2 ml of water was added. After centrifugation at 1006.2 g for 5 min, the supernatant was collected and the procedure was repeated twice with 2 ml of cyclohexane. Finally, the solvent was evaporated to dryness at 40°C under nitrogen and the residue was dissolved in 1 ml of acetonitrile and filtered (Minisart SRP 4, PTFE‐membrane 0.45 µm). PAH separation and quantification of B(a)P were performed by HPLC, and the method was validated according to Sess‐Tchotch et al. (2018). All reagents were of HPLC grade.

### 2.9 TBARS

TBARS were analyzed using the method of Lynch and Frei (1993) with slight modifications. Briefly, 1 g of sample was mixed with 100 µl of BHT 0.1 M in ethanol and 9.9 ml potassium chloride 0.15 M with a Polytron grinder (PT2500, Kinematica AG). 0.5 ml of the supernatant was mixed with 0.25 ml of thiobarbituric acid 1% (w/v) in sodium hydroxide 5 mM and 0.25 ml of trichloroacetic acid 2.8% (w/v) and incubated at 80°C for 10 min. After the mixture cooled, 2 ml of butanol was added. The absorbance of the butanol phase after centrifugation at 4 rpm for 10 min was read at 535 nm. TBARS were calculated in mg equivalent malondialdehyde (MDA) per kg of meat using a calibration curve of 1,1,3,3‐tetramethoxypropane (Sigma‐Aldrich).

### 2.10 Microbial counts

Microbial analyses were performed on selective media in duplicate. Lactic acid bacteria (LAB), coagulase‐negative staphylococci (CNS), yeasts and molds, *Enterobactericae*, coagulase‐positive staphylococci and *S. aureus*, *Listeria monocytogenes* and *Salmonella* were analyzed as described by Lebert et al. (2007). In addition, total counts were enumerated in plate count agar at 30°C for 72 hr; *Bacillus cereus* on *Bacillus cereus* selective agar at 30°C for 18–48 hr; *Clostridium perfringens* on tryptose sulfite agar at 37°C for 20 hr and confirmed with lactose sulfite medium at 46°C; and *Escherichia coli* on tryptone bile X‐glucuronide agar at 44°C for 48 hr.

### 2.11 Statistical analyses

Two‐way ANOVA was performed to check for the influence of the meat used as raw material and processing on the physicochemical and microbiological composition of kitoza using Statistica™, version 7.0 (StatSoft). Fisher's least significant difference test was used in post hoc analysis to determine difference between means with a level of significance of 95%.

## 3 RESULTS

### 3.1 Physicochemical characteristics

The physicochemical characteristics of kitoza are shown in Table 1 along with the levels of significance of the effect of the meat used as raw material (beef or pork) and processing (salted/sun‐dried, or salted/smoked).

**Table 1:** Means ± standard errors (*n* = 60) for physicochemical characteristics of kitoza and levels of significance of the effect of raw material (beef or pork) and processing (salted/sun‐dried or salted/smoked)

	Mean ± *SD*	*p*‐Level		
		Raw material	Process	Raw material × process
Moisture (g/100 g)	41.5 ± 12.1	NS	***	NS
Salt (g/100 g)	3.3 ± 1.4	NS	**	NS
Salt (g/100 g dm)	5.8 ± 2.1	NS	NS	NS
a_w_	0.893 ± 0.073	NS	***	*
Protein (g/100 g)	43.5 ± 10.8	NS	*	NS
Protein (g/100 g dm)	76.4 ± 20.6	NS	**	*
Fat (g/100 g)	14.3 ± 8.7	***	NS	NS
pH	6.04 ± 0.44	***	NS	**
Titratable acidity (meq/100 g)	10.9 ± 3.3	*	NS	NS
D‐lactic acid (g/100 g)	0.12 ± 0.16^a^ <0.014 (*n* = 15) 0.15 ± 0.17 (*n* = 45)	NS	NS	NS
L‐lactic acid (g/100 g)	0.78 ± 0.63	***	NS	**
Phenol (mg/100 g)	1.6 ± 1.7	NS	***	NS
B(a)P (µg/kg)	4.3 ± 10.6	NS	*	NS
TBARS (mg MDA/kg)	3.5 ± 3.7	NS	***	*
TBARS (mg MDA/kg dm)	5.6 ± 6.0	NS	***	NS

Abbreviations: dm, dry matter; NS: not significant.

a Values below the detection threshold (0.014 g/100 g) were considered equal to the detection threshold for calculations of means and statistical analysis.

*p*‐Level:

* *p* ≤ 0.05;

** *p* ≤ 0.01;

*** *p* ≤ 0.001.

There was no effect of the raw material (beef or pork) on the moisture and salt content and *a*_w_. However, dried kitoza had a lower moisture content than smoked kitoza (32.8 ± 8.8 and 50.3 ± 7.9 g/100 g on average, respectively). Dried kitoza samples were also on average more salted (3.9 ± 1.5 g/100 g) than smoked samples (2.8 ± 1.1 g/100 g). This was due to the effect of concentration caused by the removal of water, which was more pronounced in dried kitoza, as shown by the nonsignificant effect of processing on salt content when expressed on a dry basis. As a result, *a*_w_ of samples of dried kitoza (0.844 ± 0.065) was significantly lower than *a*_w_ of the samples of smoked kitoza (0.942 ± 0.040). Salted/sun‐dried kitoza are produced by households and are usually left hanging until consumption, and thus continue to dry which can explain these differences. There was marked variation in moisture content and *a*_w_. Moisture and *a*_w_ ranged from 13.7 to 49.3 g/100 g and from 0.646 to 0.946, respectively, in dried products. In smoked products, moisture and *a*_w_ ranged from 30.0 to 60.8 g/100 g and 0.777 to 0.977. Salt content ranged from 1.3 to 6.8 g/100 g. The moisture content and *a*_w_ of kitoza showed that salted/sun‐dried kitoza were in the range of intermediate moisture food (moisture content between 20% and 50% and *a*_w_ between 0.6 and 0.9 as defined by Leistner and Rodel (1976)). Other intermediate moisture salted/dried/smoked products include biltong, boucané, made from pieces of meat, charqui and cecina, lacón or pastirma made from whole meat muscles and dry fermented sausages made from comminuted meats (Santchurn et al., 2012). Most of the salted/smoked kitoza were in the range of high moisture food although both dried and smoked kitoza are stored at ambient temperatures in Madagascar.

There was a significant (*p* ≤ 0.05) effect of processing on protein content expressed on both a wet and dry basis, with dried kitoza showing a higher protein content (46.2 ± 11.8 g/100 g) than smoked kitoza (40.7 ± 9.0 g/100 g). The interaction between the raw material and the processing on the protein content was significant when expressed only on a dry basis. In the case of beef, there was no significant difference in protein content between the dried and smoked products when expressed on a dry basis, suggesting that the higher protein content in dried kitoza was probably due to the concentration of protein as a consequence of its lower moisture content. For pork, protein content expressed on a dry basis was significantly lower in dried kitoza (63.1 ± 22.6 g/100 g dry matter [dm]) than in smoked kitoza (86.7 ± 26.0 g/100 g dm). This reveals that the pork used to produce the smoked pork kitoza sampled in this study probably had a higher average protein content than the raw material used to make the dried pork kitoza. The fat content of the pork was higher than that of the beef kitoza (18.1 ± 9.8 and 10.5 ± 5.5 g/100 g, respectively) but lower than pork smoked boucané (49.1 g/100 g; Poligne et al., 2001).

There was a significant (*p* ≤ 0.001) effect of the meat used on pH. Beef kitoza had a lower pH (5.79 ± 0.22) than pork kitoza (6.29 ± 0.47). The main effect of processing was not significant but the interaction between the meat used and the processing revealed that pork dried products had a higher pH than smoked products (6.48 ± 0.30 and 6.09 ± 0.54, respectively). Titratable acidity was about 10 meq/100 g. They were in agreement with pH values as titratable acidity was significantly higher in beef kitoza (11.9 ± 2.8 meq/100 g) than in pork kitoza (9.9 ± 3.5 meq/100 g). No effect of processing on titratable acidity was shown. The pH of pork kitoza (6.29 on average) was similar to the pH of pork boucané (6.18) (Poligne et al., 2001) and of pork lacón (5.84–6.45; Marra, Salgado, Prieto, & Carballo, 1999). The pH of beef kitoza (5.79 ± 0.22, ranging from 5.26 to 6.22) was lower than the pH of pork kitoza but similar to the pH of salted/dried beef products such as pastirma (5.7–6.1; Kilic, 2009) and sheep kaddid (5.2–5.4; Bennani, Zenati, Faid, & Ettayebi, 1995). The pH was higher than values found in dry fermented sausages. In dry fermented sausages, the pH is reduced to values that vary from 5.5 to 4.6 during fermentation (Ockerman & Basu, 2015) due to the lactic acid bacteria that catabolize the added dextrose in D‐lactic acid reaching 0.3 to 0.7 g/100 g (Durand, 1999). D‐lactic acid content in kitoza was below the detection threshold (0.014 g/100 g) in 15 samples, and the mean value of samples above the detection threshold was 0.15 ± 0.17 g/100 g. However, seven samples had a D‐lactic acid content of more than 0.5 g/100 g. The fact that sugar is sometimes added during processing could explain these values, but confirmation would require further studies. There was no effect either of the meat used or of processing. Mean L‐lactic acid content was 0.78 ± 0.63 g/100 g with a significant (*p* ≤ 0.001) effect of the meat used, beef kitoza (1.32 ± 0.36 g/100 g) showing a higher L‐lactic acid content than pork kitoza (0.23 ± 0.27 g/100 g). Glycogen is metabolized in L‐lactic acid postmortem. Thus, lower L‐lactic acid content on average in pork kitoza is in accordance with its higher pH compared to beef. The effect of processing was not significant but the significant (*p* ≤ 0.01) interaction revealed that dried kitoza made from beef had a higher L‐lactic acid content than smoked ones (1.18 ± 0.17 and 1.46 ± 0.45 g/100 g, respectively). However, the difference was too small to affect pH.

Phenol content of smoked kitoza was 2.8 ± 1.8 mg/100 g. Among dried samples, 22 samples had a phenol content higher than 0.1 mg/100 g and 4 (two of each type of meat) had a phenol content of the order of 1 mg/100 g. There was no effect of the kind of meat used as raw material (beef or pork). Salted/smoked kitoza had a much higher phenol content (2.8 ± 1.8 mg/100 g on average) than kundi (0.5 to 14.4 mg/100 g) but similar to that of boucané (3.0 mg/100 g; Alonge, 1987; Poligne et al., 2001). B(a)P content of smoked kitoza was 7.8 ± 13.8 µg/kg on average and reached 59 µg/kg. There are no regulations concerning PAH content of smoked meat products in Madagascar. However, 11 out of 30 smoked samples and two out of 30 dried samples had a B(a)P content higher than the limit of 2 µg/kg set in European regulations. Surprisingly, some samples of salted/sun‐dried kitoza were contaminated by small amounts of phenols and B(a)P. This could be explained by the fact that kitoza is dried in the sun during the day and hung in the kitchen where there is a fire during the night.

The mean TBARS index was 3.5 ± 3.7 mg MDA/kg. They differed significantly depending on the type of processing. Smoked kitoza had a lower TBARS index than dried kitoza (1.4 ± 2.5 and 5.6 ± 3.7 mg/kg, respectively). This was not due to their higher water content as the effect was also apparent in TBARS expressed on a dry basis; neither can it be related to the fat contents which did not differ significantly in the smoked and dried samples. Literature on lipid oxidation of salted/dried/smoked or roasted meat products made of noncomminuted beef and pork meat products includes tsire and kilishi, salted/dried/roasted beef meat products in western Africa and charqui (salted/dried beef). Igene, Farouk, and Akanbi (1990) reported an increase in TBARS from 1.5 to 2 mg MDA/kg in kilishi during 60 weeks of storage, and Onilude, Sanni, Olaoye, and Ogunbanwo (2002) reported TBARS to be less than 0.7 mg MDA/kg after roasting. For dried products, values ranging from less than 2.29 mg MDA/kg have been reported in pastirma (Abdallah, Mohmaed, Mohamed, & Emara, 2017; Aksu & Kaya, 2002) while higher values (4.5 mg MDA/kg) were reported in beef charqui after drying (Torres, Shimokomaki, Franco, & Landgraf, 1994). Salted/smoked kitoza had lipid oxidation values (TBARS) (1.4 ± 2.5 mg/kg) similar to those reported for tsire and kilishi. TBARS values of salted/sun‐dried kitoza (5.6 ± 3.7 mg/kg) were much higher than values reported for other salted/dried meat products like pastirma and charqui. The higher salt content of dried kitoza could explain the higher TBARS values of dried kitoza compared to the smoked ones due to the prooxidant effect of salt (Mariutti & Bragagnolo, 2017). Moreover, the antioxidant effect of phenols (Sikorski & Sinkiewicz, 2015) probably contributed to the lower TBARS values in smoked kitoza.

### 3.2 Microbial counts

Microbial counts of kitoza and levels of significance of the effect of the meat used (beef or pork) and processing (salted/sun‐dried or salted/smoked) are shown in Table 2.

**Table 2:** Means ± standard errors (*n* = 54) of microbiological count of kitoza and levels of significance of the effect of raw material (beef or pork) and processing (salted/sun‐dried or salted/smoked)

	Mean ± *SD* (log cfu/g)	*p*‐Level		
		Raw material	Process	Raw material × process
Total count	7.0 ± 1.3	**	NS	NS
LAB	6.8 ± 1.3	**	NS	NS
CNS	6.4 ± 1.5	*	**	NS
Enterobacteriaceae	2.0 ± 1.3^a^ <1 (*n* = 26) 3.0 ± 1.2 (*n* = 28)	NS	***	NS
*Escherichia coli*	1.9 ± 1.4^a^ <1 (*n* = 29) 3.0 ± 1.4 (*n* = 25)	***	***	***
*Bacillus cereus*	<2.0 (*n* = 54)	—	—	—
*Staphylococcus aureus*	2.8 ± 1.0^a^ <2 (*n* = 29) 3.4 ± 1.0 (*n* = 25)	NS	***	NS
*Listeria monocytogenes*	Absent in 25 g (*n* = 53) Present in 25 g (*n* = 1)	—	—	—
*Salmonella*	Absent in 25 g (*n* = 54)	—	—	—
*Clostridium perfringens*	<1 (*n* = 54)	—	—	—
Y/M	3.7 ± 1.7^a^ <1 (*n* = 5) 4.0 ± 1.5 (*n* = 49)	***	***	NS

Abbreviations: CNS, coagulase‐negative staphylococci; LAB, lactic acid bacteria; NS, not significant; —, not adapted; Y/M, yeast and mold.

a Values below the detection threshold were considered equal to the detection threshold for calculations of means and statistical analysis.

*p*‐Level

* *p* ≤ 0.05;

** *p* ≤ 0.01;

*** *p* ≤ 0.001.

Total counts were on average 7.0 ± 1.3 log cfu/g, probably linked with the high counts of LAB and CNS (on average 6.8 ± 1.3 and 6.4 ± 1.5 log cfu/g, respectively). The effect of the raw material was significant with higher total counts in pork kitoza (7.5 ± 1.1 log cfu/g) than in beef kitoza (6.5 ± 1.4 log cfu/g) as well as for LAB (7.3 ± 1.0 and 6.3 ± 1.4 log cfu/g in pork and beef, respectively) and CNS (6.8 ± 1.4 and 6.0 ± 1.0 log cfu/g in pork and beef, respectively). For CNS, processing also had a significant effect (*p* ≤ 0.01). CNS were higher in dried products than in smoked products (6.9 ± 1.0 and 5.9 ± 1.7 log cfu/g, respectively).

**Figure 1:**
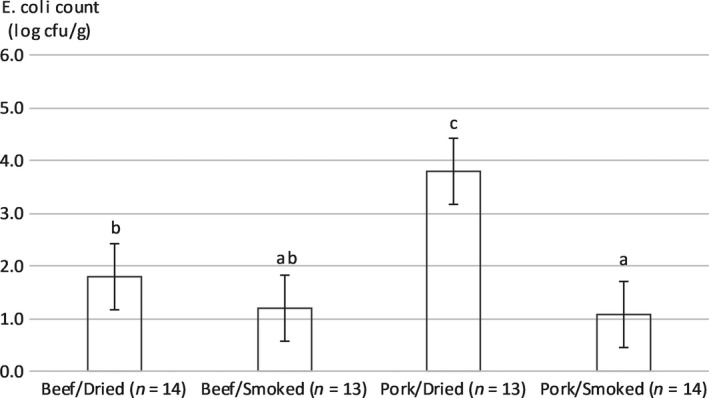
*Escherichia coli* counts for kitoza as a function of the raw material (beef or pork) and type of processing (salted/sun‐dried or salted/smoked). Error bars represent standard deviation. Values below the detection threshold were considered equal to the detection threshold for calculation of means and statistical analysis

Enterobacteriaceae and *E. coli* are considered indicators of fecal contamination. Regarding *E. coli*, 29 samples were below the detection threshold (<1 log cfu/g). For the 25 samples above the detection threshold, the count was on average 3.0 ± 1.4 log cfu/g and for 18 samples, counts were above the recommended level (<2 log cfu/g) for smoked products (bacon) as defined in European Regulation 2073/2005 (European Commision, 2005). The main effects of the raw material and of processing and their interaction were significant (*p* ≤ 0.001). Pork kitoza had higher counts than beef kitoza (2.4 ± 1.6 and 1.5 ± 0.9 log cfu/g, respectively). Dried products had higher counts than smoked products but only in pork kitoza (3.8 ± 1.3 and 1.1 ± 0.2 log cfu/g, respectively; Figure 1). Concerning Enterobacteriaceae, 26 samples were below the detection threshold (1 log cfu/g). For the samples above the detection threshold (*n* = 28), the count was 3.1 ± 1.2 log cfu/g on average and 5 samples had counts above the recommended level (<4 log cfu/g) for smoked products (bacon) as defined in European Regulation 2073/2005 (European Commission, 2005). Our results showed that dried kitoza had higher counts than smoked kitoza (2.7 ± 1.4 and 1.4 ± 1.0 log cfu/g, respectively). These results showed that products were mostly of good hygienic quality but that dried kitoza was of lower hygienic quality (with higher *E. coli* counts for the pork products and higher Enterobacteriaceae counts in both pork and beef products).

Microbial analyses revealed the absence of pathogenic bacteria such as *Salmonella*, and only 1 sample out of 54 was contaminated with *L. monocytogenes*. Furthermore, *Bacillus cereus* and *Clostridium perfringens* were all below the detection threshold (2 and 1 log cfu/g respectively). *S. aureus* was below the detection threshold (2 log cfu/g) in 25 samples and in average of 3.3 ± 1.0 log cfu/g when detected, and 21 out of 29 samples were above the 2.7 log cfu/g level tolerated for smoked products (bacon) as defined in European Regulation 2073/2005 (European Commission, 2005). There was a significant effect of processing (*p* ≤ 0.001) with higher *S. aureus* counts in dried kitoza (3.4 ± 1.0 log cfu/g) than in smoked ones (2.3 ± 0.7 log cfu/g).

Yeasts and molds were on average 4.0 ± 1.5 log cfu/g in the 49 samples above the detection threshold (2 log cfu/g). Both raw material and processing affected significantly their level (*p* ≤ 0.001). Counts were higher in pork kitoza (4.5 ± 1.6 log cfu/g) compared to beef ones (2.9 ± 1.4 log cfu/g) and in dried ones (4.4 ± 1.5 log cfu/g) compared to smoked ones (3.0 ± 1.5 log cfu/g). Yeasts and molds have the ability to degrade L lactate (Selgas & Garcia, 2015) which could explain the low titratable acidity in some dry fermented sausages.

Lactic acid bacteria and CNS were predominant in kitoza as in meat products such as cecina, charqui, and pastirma (about 7 log cfu/g) (Garcia, Zumalcarregui, & Diez, 1995; Kaban, 2009; Pinto, Ponsano, Franco, & Shimokomaki, 2002) and in most traditional fermented sausages (Leroy, Lebert, & Talon, 2015). LAB and CNS were probably favored by the process used (salting, drying). In fact, CNS were higher in dried pork kitoza. Significant differences between the distribution of CNS species were also observed in a previous study (Ratsimba et al., 2017). The high total counts measured in kitoza (on average 7 log cfu/g) were related to the high counts of LAB and CNS. Similar total counts have been reported on biltong (Jones et al., 2017), kilishi (about 7 log cfu/g) (Mbawala, Daoudou, & Ngassoum, 2010), and pastirma (4–8 log cfu/g) (Kilic, 2009) sampled on markets and in some traditional dry fermented sausages (Leroy et al., 2015). Lower total counts (4–6 log cfu/g) have been reported in charqui (Pinto et al., 2002) and kaddid (Bennani et al., 1995).

Although salted/sun‐dried kitoza had lower *a*_w_, they had higher counts of *E. coli*, Enterobacteriaceae, *S. aureus,* and yeasts and molds. The lowest counts in salted/smoked kitoza could be related to the high temperature probably reached during smoking and to the properties of phenols from the smoke. Moreover, it can be postulated that the processing and storage conditions of salted/sun‐dried kitoza, usually processed at the household level where they are dried in the open air and stored until consumption, could be related to their higher microbial counts. The low levels of pathogens were probably related to the combination of drying, smoking, and curing methods during processing. Enterobacteriaceae, *S. aureus*, or yeasts and molds counts of the same order have also been reported in other traditional meat products (Bennani et al., 1995; Kilic, 2009; Leroy et al., 2015; Lorenzo et al., 2015; Mbawala et al., 2010).

## 4 CONCLUSION

Microbial properties of kitoza revealed that, although good hygienic practices are sometimes lacking, as shown by *E. coli* counts, kitoza was not contaminated by the majority of possible pathogens, except *S. aureus* which was found on occasion and with higher counts in salted/sun‐dried kitoza than in salted/smoked kitoza. LAB and CNS were the predominant flora. While in dried kitoza, *a*_w_ was in the same range as in intermediate moisture food (*a*_w_ < 0.9), most of the smoked kitoza had a higher *a*_w_ which is not in accordance with their storage at ambient temperatures as done in Madagascar. Stability of smoked kitoza could be increased by a higher reduction in moisture content during smoking. But if there is no change in the smoking monitoring, this could result in even higher B(a)P content, which is already above the limits laid down in countries where these are the subject of regulation. Moreover, reducing moisture content may not be appreciated by consumers. Another solution, such as the addition of bioprotective cultures and the reduction of pH, could improve the microbial stability of kitoza.

## CONFLICT OF INTEREST

The authors declare no conflict of interest.

## ETHICAL STATEMENT

This study does not involve any human or animal testing.
